# Hydrogen Peroxide Is a Second Messenger in the Salicylic Acid-Triggered Adventitious Rooting Process in Mung Bean Seedlings

**DOI:** 10.1371/journal.pone.0084580

**Published:** 2013-12-27

**Authors:** Wei Yang, Changhua Zhu, Xiaoling Ma, Guijun Li, Lijun Gan, Denny Ng, Kai Xia

**Affiliations:** 1 Laboratory of Plant hormone, College of Life Sciences, Nanjing Agricultural University, Nanjing, China; 2 CP Bio, Inc., Chino, California, United States of America; National Taiwan University, Taiwan

## Abstract

In plants, salicylic acid (SA) is a signaling molecule that regulates disease resistance responses, such as systemic acquired resistance (SAR) and hypertensive response (HR). SA has been implicated as participating in various biotic and abiotic stresses. This study was conducted to investigate the role of SA in adventitious root formation (ARF) in mung bean (*Phaseolus radiatus* L) hypocotyl cuttings. We observed that hypocotyl treatment with SA could significantly promote the adventitious root formation, and its effects were dose and time dependent. Explants treated with SA displayed a 130% increase in adventitious root number compared with control seedlings. The role of SA in mung bean hypocotyl ARF as well as its interaction with hydrogen peroxide (H_2_O_2_) were also elucidated. Pretreatment of mung bean explants with N, N’-dimethylthiourea (DMTU), a scavenger for H_2_O_2_, resulted in a significant reduction of SA-induced ARF. Diphenyleneiodonium (DPI), a specific inhibitor of membrane-linked NADPH oxidase, also inhibited the effect of adventitious rooting triggered by SA treatment. The determination of the endogenous H_2_O_2_ level indicated that the seedlings treated with SA could induce H_2_O_2_ accumulation compared with the control treatment. Our results revealed a distinctive role of SA in the promotion of adventitious rooting via the process of H_2_O_2_ accumulation. This conclusion was further supported by antioxidant enzyme activity assays. Based on these results, we conclude that the accumulation of free H_2_O_2_ might be a downstream event in response to SA-triggered adventitious root formation in mung bean seedlings.

## Introduction

Roots function as the interface between plants and the terrestrial environment. In higher plants, the root system is composed of primary roots, lateral roots and adventitious roots. Primary roots are initiated during embryogenesis and elongate after germination. Compared with primary roots, lateral roots and adventitious roots are ‘post-embryonic’ roots, as they initiate from non-pericycle tissues. Lateral roots initiate from primary roots or axes, and adventitious roots initiate from stem and leaf-derived cells. Together, these plant roots form the root system, the architecture of which can be altered in response to environmental changes and stimuli [1].

The appearance of adventitious roots may date the evolution of endogenous initiation combined with reverse auxin transport because these roots appear to have evolved repeatedly over time, and it is suggested they may have been required for the establishment of vascular continuity [2]. Adventitious root formation (ARF) is part of the normal development of plants and occurs naturally. ARF is useful for facilitating the uptake of water and nutrients from the soil, the anchorage of plants to substrates and the formation of food storage reserves. 

ARF is a complex process that is influenced by multiple exogenous and endogenous factors. Plant hormones are among the internal factors that play a major role in regulating ARF. The hormone auxin has been shown to be a key regulator of AR formation [3-8]. Furthermore, ethylene [9-11] and cytokinin [12,13] are also believed to be crucial for ARF. In addition, other molecules such as polyamines [14], peroxidase (POD) [15], H_2_O_2_ [16,17], Ca^2+^ [18,19], nitric oxide (NO) [20-22], cyclic guanosine monophosphate (cGMP) [21,22] and mitogen-activated protein kinase (MAPKs) [23], as well as light [24], play a pivotal role in the adventitious rooting process. Although a variety of components participate in the regulation of the adventitious rooting process, the molecular and biochemical mechanisms underlying the signal transduction involved in this process remain poorly understood.

The plant hormone SA is an endogenous growth regulator with a phenolic nature that plays a critical role in many diverse physiological processes. A well-known effect of SA is increasing the temperature in thermogenic plants [25] and flowering [26,27]. Previous studies also support the notion that SA is involved in modulating the plant response to many abiotic and biotic stresses, such as disease resistance [28,29] photosynthesis [30,31], low temperature resistance[32,33], drought resistance [34,35], salt resistance [36,37] and fruit maturity [38], among others. SA is also known to play a possible role in activating the defense response of plants to pathogen attack. SA mediates the oxidative burst that leads to programmed cell death (PCD) in the hypersensitive response, and it has been suggested that SA could act as a signal in the development of the SAR [39,40]. 

Nevertheless, defining the biological functions of SA in the context of the adventitious rooting process has been controversial. Still and Kling advocated that supplying SA could slight stimulate adventitious root initiation [41,42]. And in combination with indoleacetic acid (IAA), SA act synergistically effect [42]. This study revealed a distinctive role for SA in IAA-induced adventitious root formation. In 1989, Riov found that SA could effectively stimulate rooting of mung bean cuttings. However, only two concentration of SA has been examined (0.1 mM, 0.2 mM) [5]. Using SA deficient mutants, Gutierrez suggested that SA is possibly a positive regulator of adventitious rooting in *Arabidopsis* [43]. On the other side, Kang found that SA could increase the amount of scopolamine in adventitious root cultures of *Scopolia parviflora*, and without any negative effects on growth [44]. In contrast, another study yielded completely different results. Li reported that SA inhibited adventitious root formation and decreased the weights of roots in mung bean hypocotyls cuttings [45]. And De Klerk indicated that SA inhibited IAA-induced adventitious root formation in apple microcuttings during the initial post-treatment days. These findings may be the result of SA-enhanced IAA decarboxylation [46]. To date, the confirmatory role of SA in ARF is still ambiguous. 

The main object of this study was to identify the role of SA in ARF in mung bean hypocotyls. Through pharmacological and physiological approaches, we demonstrate that SA is an important factor that induces adventitious root organogenesis and formation in mung bean hypocotyls. We also examine the interaction between SA with another important second signaling molecule, H_2_O_2_. Furthermore, we provide evidence that SA can elevate H_2_O_2_ levels through the regulation of the antioxidant enzymes in mung bean seedlings. Based on these results, a simple transduction pathway model is proposed, wherein H_2_O_2_ acts as a downstream messenger in the SA-mediated signaling pathway that induces ARF.

## Materials and Methods

### Plant Material

Mung bean (*Phaseolus radiatus* L) seeds were washed in distilled water and immersed in 0.1% HgCl_2_ for 5 minutes. After five washes in distilled water, the seeds were soaked in distilled water for 12 h at 27°C and were then maintained at 27°C for 5 d, with a 14-h photoperiod (PAR of 200 µmol m^−2^ s^−1^). 

### Explant Treatments

The explants consisted of a terminal bud, two primary leaves and 4 cm of the hypocotyls. After the primary roots were removed, the explants were put into 100-ml beakers containing 50 ml of distilled water (control) or 50 ml of the test solution for 24 h under the same conditions. After being washed three times, the seedlings were moved into distilled water, used as explants and maintained under the same temperature and photoperiod conditions for another five days. The distilled water was replaced daily.

### Application of plant hormones and inhibitors

All chemicals were purchased from Sigma-Aldrich (St Louis, USA). SA was dissolved in ethanol for a final stock concentration of 1 M. The H_2_O_2_ scavenger DMTU was dissolved in distilled water to make a stock solution of 10 M. The NADPH oxidase inhibitor DPI was dissolved in dimethylsulfoxide (DMSO) to make a stock solution of 1 mM. For the DMTU and DPI treatments, after the removal of the primary roots, mung bean seedlings were pretreated in beakers for 4 h in the presence of DMTU or DPI and were then moved into test solutions for another 24 h. In addition, all of the explants treated with the inhibitors tested in this study appeared healthy.

### Statistical analyses

The numbers of adventitious roots were determined after five days of treatment. The number of adventitious roots of more than 1 mm long was recorded. The data presented are the means of at least three independent experiments with 30 explants per treatment. The data were analyzed using an ANOVA. The comparisons between the mean values were conducted using the least signiﬁcant difference (LSD) test, with the significance set at P < 0.05, and the standard error (S.E.) was calculated. The statistical analyses were performed using SPSS software version 14.0 (SPSS Inc., Chicago, IL).

### Root primordium observation

Root primordium observation was accomplished using Feulgen-staining, as described by Kordan [47]. After being treated for 48 h, the hypocotyls cut from the explants were fixed in absolute ethanol:glacial acetic acid (3:1) for 48 h and were then stored in 70% ethanol until they were required for use. The hypocotyls were rehydrated in an ethanol series (70-50-30-10%), hydrolyzed for 10 min in 10% HCl at 60°C and then placed in Schiff’s reagent (24 h) followed by a thorough washing with tap water until the wash water was no longer pink. The Feulgen-stained hypocotyls were placed in a 10% aqueous glycerine solution in uncovered plastic Petri dishes, and the glycerine allowed for concentration via the gradual evaporation of the water on a warming plate at 40°C. The stained hypocotyls were examined as whole mounts in the concentrated glycerine in Petri dishes using bright field transmission optical microscopy.

### Visualization of H_2_O_2_ with the DAB method

H_2_O_2_ was detected with the DAB method [48]. Briefly, the hypocotyls cut from the explants were treated with 1 mg/ml solution of DAB, pH 3.8, for 8 h under light at 25°C. The treated hypocotyls were then placed in boiling 95% ethanol for 10 min to decolorize the hypocotyls, except for the deep brown polymerization product produced by the reaction of DAB with H_2_O_2_. After cooling, the hypocotyls were extracted with fresh ethanol and preserved at 4°C in ethanol and photographed.

### Cytochemical detection of H_2_O_2_

H_2_O_2_ was visualized at the subcellular level using CeCl_3_ for localization [49]. Electron-dense CeCl_3_ deposits are formed in presence of H_2_O_2_ and are visible via transmission electron microcopy. Tissue samples (2×2 mm^2^) were excised from the hypocotyls of the cuttings and then vacuum infiltrated with freshly prepared 5 mM CeCl_3_ in 50 mM 3-(N-morpholino) -propane-sulfonic acid at pH 7.2 for 30 min. The tissue samples were then fixed in 1.25% (v/v) glutaraldehyde and 1.25% (v/v) paraformaldehyde in 50 mM sodium cacodylate buffer (CAB), pH 7.2, for 1 h at room temperature and kept overnight at 4°C. After fixation, the tissue samples were washed twice for 10 min in CAB and postfixed for 45 min in 1% (v/v) osmium tetroxide in CAB. The tissue samples were then washed twice for 10 min in CAB and dehydrated in a graded acetone series (30, 50, 70, 80, 90 and 100% [v/v]), progressively embedded in rising concentrations of acetone-resin mixtures, and incubated over two 24 h replacements of pure epoxy resin before polymerization at 60°C for 48 h. Ultrathin sections (50 to 100 nm) were obtained with a Reichert Ultracut E Microtome, using a diamond knife mounted on nickel grids (200 mesh), and examined without staining with a transmission electron microscope (H7650, HITACHI, Japan) at an accelerating voltage of 80 kV.

### Endogenous H_2_O_2_ assay

H_2_O_2_ content was measured by monitoring the A_415_ of the titanium-peroxide complex, following the method described by Brennan and Frenkel [50], with slight modifications. Briefly, 0.5 g (FW) of hypocotyl material was frozen in liquid nitrogen immediately after completion of the treatment period and ground with liquid nitrogen, and the ﬁne powdered material was mixed with 5 ml of cooled acetone in an ice bath. The mixture was centrifuged at 10,000 g for 15 min (4°C). Next, 1 ml of supernatant was obtained, and this was followed by the addition of 0.2 ml of a titanium reagent (20% w/v) and 0.4 ml of an ammonium solution to precipitate the titanium-hydroperoxide complex. The reaction mixture was centrifuged at 10,000 g for 10 min. The precipitate was dissolved in 5 ml of 2 M H_2_SO_4_. The supernatant’s absorbance was measured at 415 nm against blanks using a LabTech UV1600 spectrophotometer (LabTech Inc. USA). A standard response curve was prepared with known concentrations of H_2_O_2_ using the same method as described above. The H_2_O_2_ content was calculated through comparison with a standard graph generated with known H_2_O_2_ concentrations.

### Determination of hypocotyl O_2_^-^ production

O_2_^-^ production was measured, as described by Able [51], by monitoring the reduction of XTT in the presence of O_2_^-^. Mung bean hypocotyls (1 g) were homogenized with 5 ml of 50 mM Tris-HCl buﬀer (pH 7.5) and centrifuged at 15,000 g for 10 min. The reaction mixture (1 ml) contained 50 mM Tris–HCl buﬀer (pH 7.5), 50 μg of plasma membrane proteins, and 0.5 mM XTT. The reduction of XTT was evaluated at 470 nm for 5 min. Corrections were made for the background absorbance in the presence of 50 units of SOD. The O_2_^-^ production rate was calculated using an extinction coefficient of 2.16×10^4^ M^–1^ cm^–1^.

### Isolation of the plasma membrane and determination of NADPH oxidase activity in the plasma membrane

Plasma membrane vesicles were isolated from mung bean hypocotyls using two-phase partitioning, according to a procedure described previously [52]. The membrane vesicles were resuspended in a 50 mM Tris-HCl buffer, pH 7.5 and used immediately for NADPH oxidase activity assays. The membrane vesicles (10 μg) were incubated in the reaction buffer (50 mM Tris-HCl buffer, pH 7.5, and 0.5 mM XTT). The reaction was initiated with the addition of NADPH. After 10 min at 25°C, the reaction solution was used for the spectrophotometric analysis of XTT formazan production at A_470_. The NADPH activity was expressed as △A_470_ per milligram of protein per minute. △A_470_ represents the difference in XTT formazan absorbance at 470 nm in the presence and absence of 100 units of SOD.

### Enzyme activity assays

For the determination of enzyme activity, plant material was frozen in liquid N_2_ and stored at -80°C until the plants were ground and enzymes were extracted. Frozen root segments (0.3 g) were homogenized in 5 ml of 50 mM potassium phosphate buﬀer (pH 7.0) containing 1 mM EDTA and 1% polyvinylpyrrolidone, with the addition of 1 mM ASC in the case of the APX assay. The homogenate was centrifuged at 15,000 g for 20 min at 4°C, and the supernatant was immediately used for the following described enzyme assays. Protein content was determined, according to the Bradford method, with BSA as a standard.

SOD activity was assayed by monitoring the inhibition of the photochemical reduction of nitro blue tetrazolium (NBT), according to the method of Giannopolitis and Ries [53]. One unit of SOD activity was deﬁned as the amount of enzyme that was required to produce 50% inhibition of the reduction of nitro blue tetrazolium as monitored at 560 nm. 

CAT activity was assayed by measuring the rate of decomposition of H_2_O_2_ at 240 nm, as described by Aebi [54]. 

GR activity was determined, as described by Connell and Mullet [55]. The activity of the enzyme was assessed in NADPH-containing HEPES buffer by monitoring the decrease in absorbance at 340 nm as NADPH is oxidized. 

APX activity was determined by following the decrease in 290 nm for 3 min in a reaction mixture containing 50 mM potassium phosphate buffer (pH 7.0), 0.5 mM ASC, 0.1 mM H_2_O_2_, and 200µl of enzyme extract. The reaction was started by the addition of the enzyme extract. The reaction was corrected for the low non-enzymatic oxidation of ASC by H_2_O_2_ [56].

## Results

### SA induces adventitious rooting in mung bean hypocotyls in a dose- and time-dependent manner

**Figure 1:**
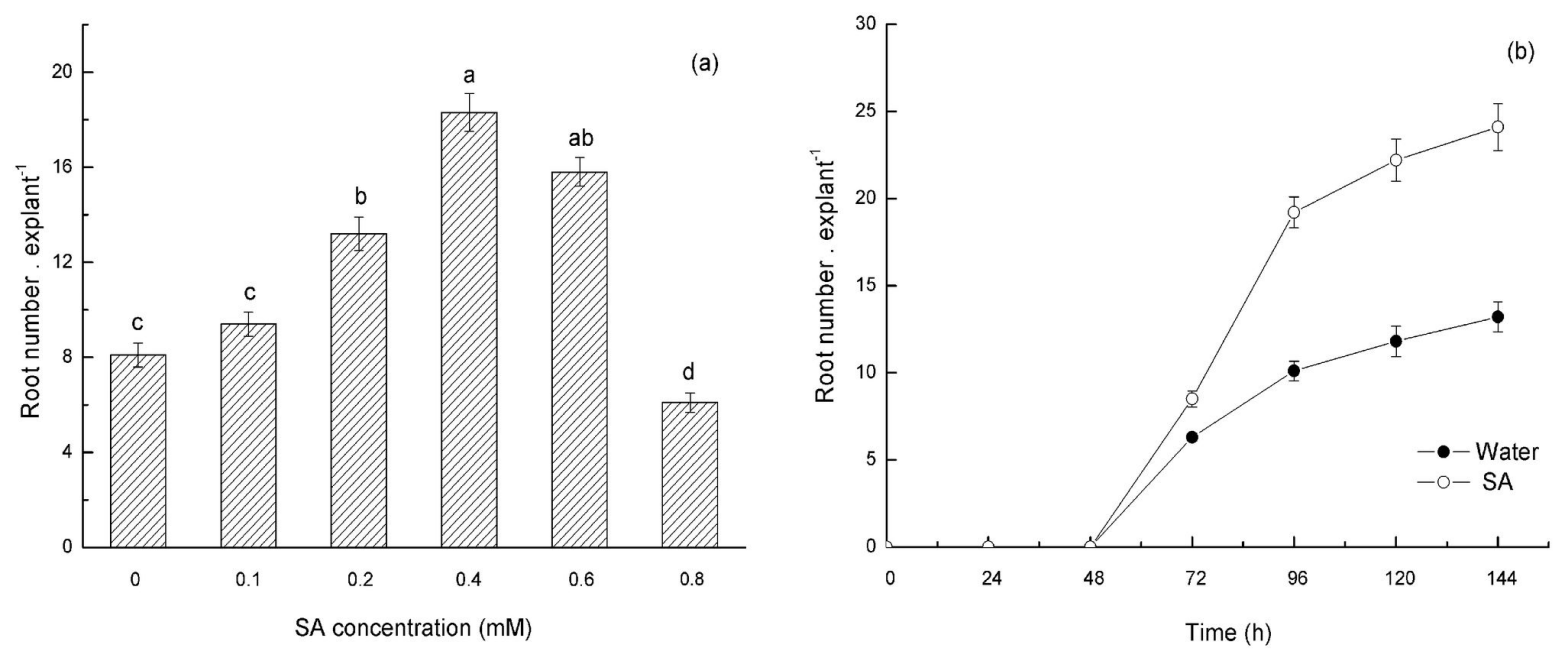
SA-induced ARF increases in mung bean hypocotyl cuttings. (a) Hypocotyls were incubated with 0, 0.1, 0.2, 0.4, 0.6 and 0.8 mM SA for 24 h and washed three times, and then the cuttings were transferred into distilled water. The cuttings were continuously grown for 5 days in this distilled water at 25±2°C, with a 14-h photoperiod (PAR of 200 µmol m^−2^ s^−1^). The distilled water was replaced every day. The root numbers were determined at 5 d after treatment. In addition, adventitious roots of more than 1 mm long were quantified. The values represent the means of 30 explants, and the different letters above the bars indicate significant differences among the treatments at a P<0.05 level, according to the LSD test. (b) Time course of adventitious root formation induced by application of SA or water in mung bean hypocotyls. Hypocotyls were treated with 0.4 mM SA (the optimal concentration) or CK (water) for 24 h and were then transferred into distilled water and continuously grown for 7 days at 25±2°C, with a 14-h photoperiod (PAR of 200 µmol m^−2^ s^−1^). The root numbers were determined in 24-h intervals. The distilled water was replaced every day, and the number of adventitious roots of more than 1 mm long was recorded. The values represent the means of 30 explants, and the error bars represent the SE (P<0.05) .

To investigate the effect of SA on ARF in mung bean hypocotyls, 5-d-old seedlings with their primary roots removed were used as explants and incubated in a control treatment (water) and different concentrations of SA (0.1, 0.2, 0.4, 0.6 and 0.8 mM) at the same temperature and with the same photoperiod conditions for 24 h. After 5 days, the root number was analyzed and quantiﬁed. The results indicated that exogenous SA could promote adventitious root formation, and its effects were dose and time dependent (Figure 1). The treatment of the seedling explants with a low concentration of SA (0.2 mM) slightly promoted ARF, and the root number of treatment explants exposed to 0.4 mM SA was 2.3-fold compared with the explants in the control treatment. An inhibitory effect on ARF was observed when the SA concentration was 0.8 mM. These results indicate that exogenous SA at particular concentrations promotes adventitious root formation in mung bean hypocotyl cuttings. Therefore, an SA concentration of 0.4 mM was used in the further experiments.

### Effect of SA on root primordia formation

**Figure 2:**
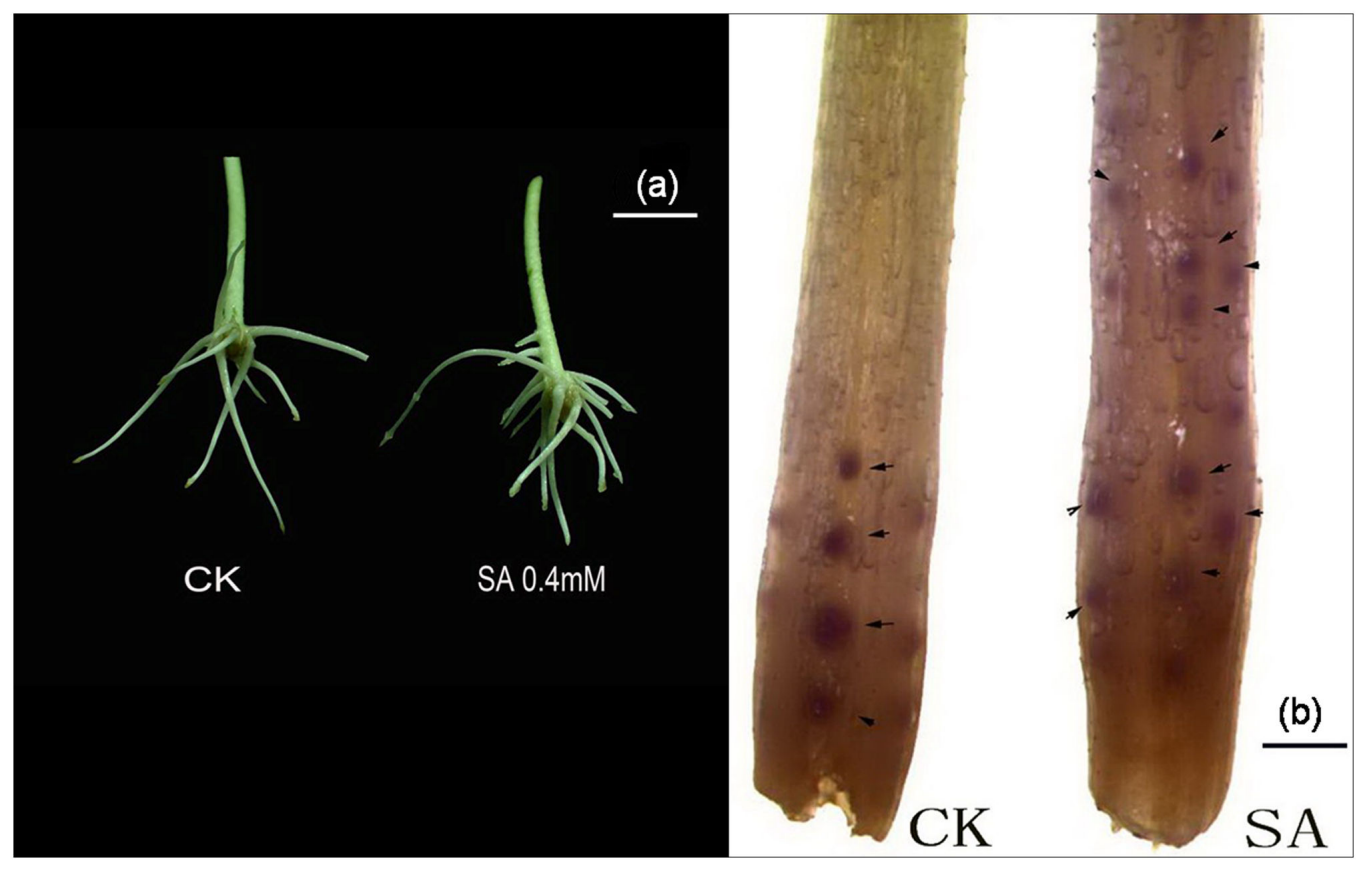
(a) Photograph showing explants after 5 days of treatment with CK (water) and SA (0.4 mM) Bar=1 cm. (b) Photograph depicting adventitious root primordial formation in mung bean seedlings after 48 h of treatment with H_2_O or 0.4 mM SA. Bar=1 mm.

An anatomical study was performed to observe the primordium formation during the first stages of adventitious rooting. We analyzed (0-1 cm) sections of mung bean hypocotyls treated with water (control) or SA (0.4 mM SA). A photograph of a representative root primordium observation is presented in Figure 2b. At 48 h after the removal of the primary root system, adventitious root primordium formation was detected in explants treated with SA and water. In the water-treated plants, few root primordia could be observed, whereas in the SA-treated explants, we observed a greater number of root primordia than in the water-treated hypocotyls. The number of root primordia in the SA-treated explants was more than 2-fold higher than in the control seedlings. These results suggested that SA promotes AR formation in mung bean hypocotyls, possibly due to the induction of the dedifferentiation of cells and the consequent reestablishment of a new apical meristem.

### Interaction between SA and H_2_O_2_ in ARF in mung bean hypocotyls

**Figure 3:**
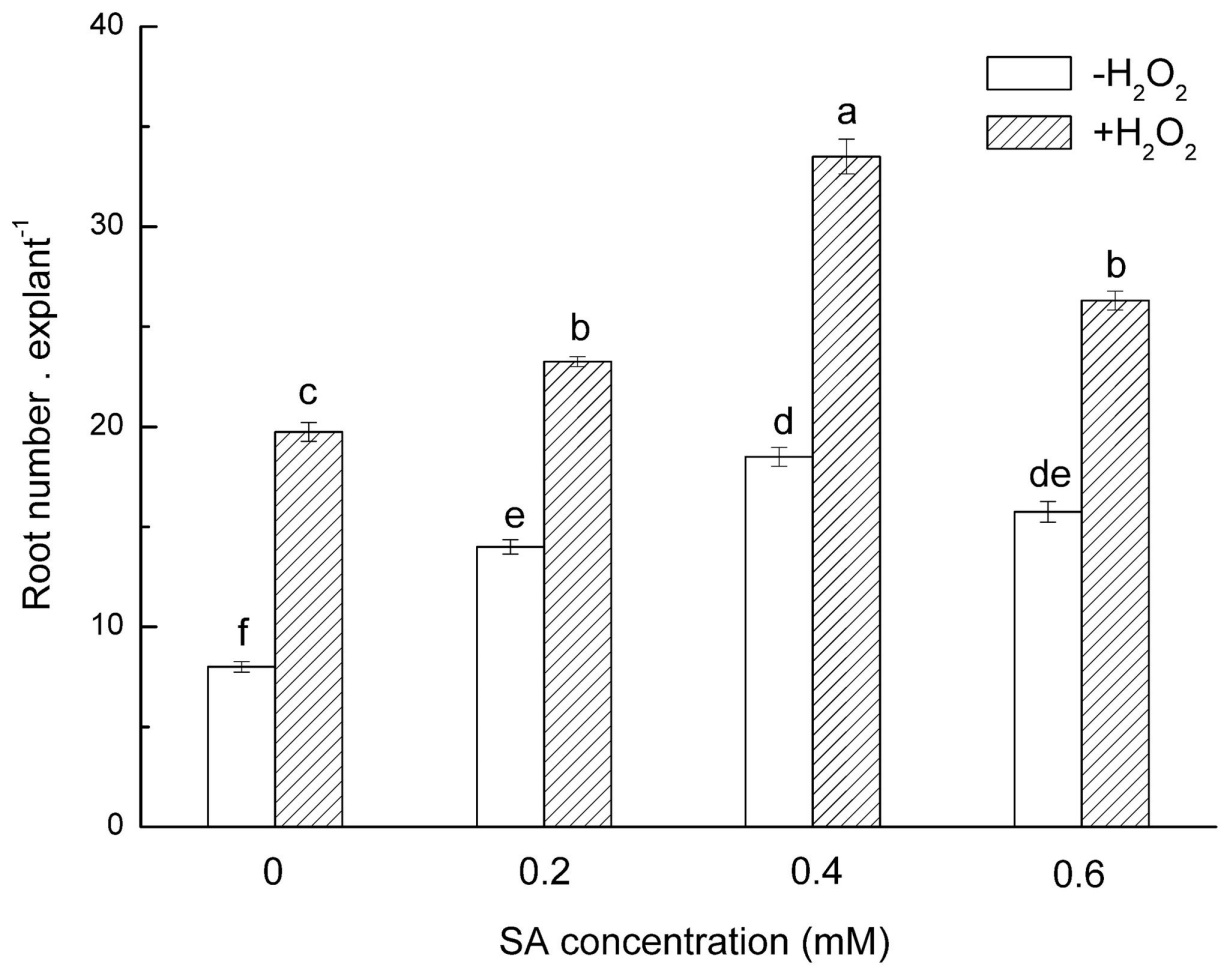
Interaction between SA in combination with H_2_O_2_ on ARF in mung bean hypocotyls. Hypocotyls were treated with different test solutions for 24 h, and then the cuttings were transferred to distilled water and continuously grown for 5 days at 25±2°C, with a 14-h photoperiod (PAR of 200 µmol m^−2^ s^−1^). The distilled water was replaced every day, and the number of adventitious roots of more than 1 mm long was recorded. The number of roots was determined after 5 d of treatment. The values represent the means of 30 explants, and the error bars represent the SE (P<0.05). H_2_O_2_:10 mM H_2_O_2_.

The above data indicate a SA-dependent effect on the formation of adventitious roots in mung bean hypocotyls. Previous work has shown that H_2_O_2_ can stimulate ARF in mung bean hypocotyls, and H_2_O_2_ might act as a downstream signaling molecule in IAA-induced ARF [57-59]. Whether SA influences H_2_O_2_-induced ARF remains unknown. To investigate whether H_2_O_2_ acts as a messenger in the SA-induced ARF transduction pathway, we first investigated the influence of SA on H_2_O_2_-induced ARF in mung bean hypocotyls. Several concentrations of SA with low concentrations of H_2_O_2_ (10 mM, suboptimal concentration) were tested together. We found that a combination of SA and H_2_O_2_ plays a synergetic role in regulating adventitious rooting in mung bean hypocotyls. At low concentrations, supplying SA with H_2_O_2_ can promote their respective effects on ARF in mung bean hypocotyls, whereas at a higher concentration, SA and H_2_O_2_ suppressed ARF (Figure 3). 

### DMTU and DPI prevent SA-induced adventitious root formation

**Figure 4:**
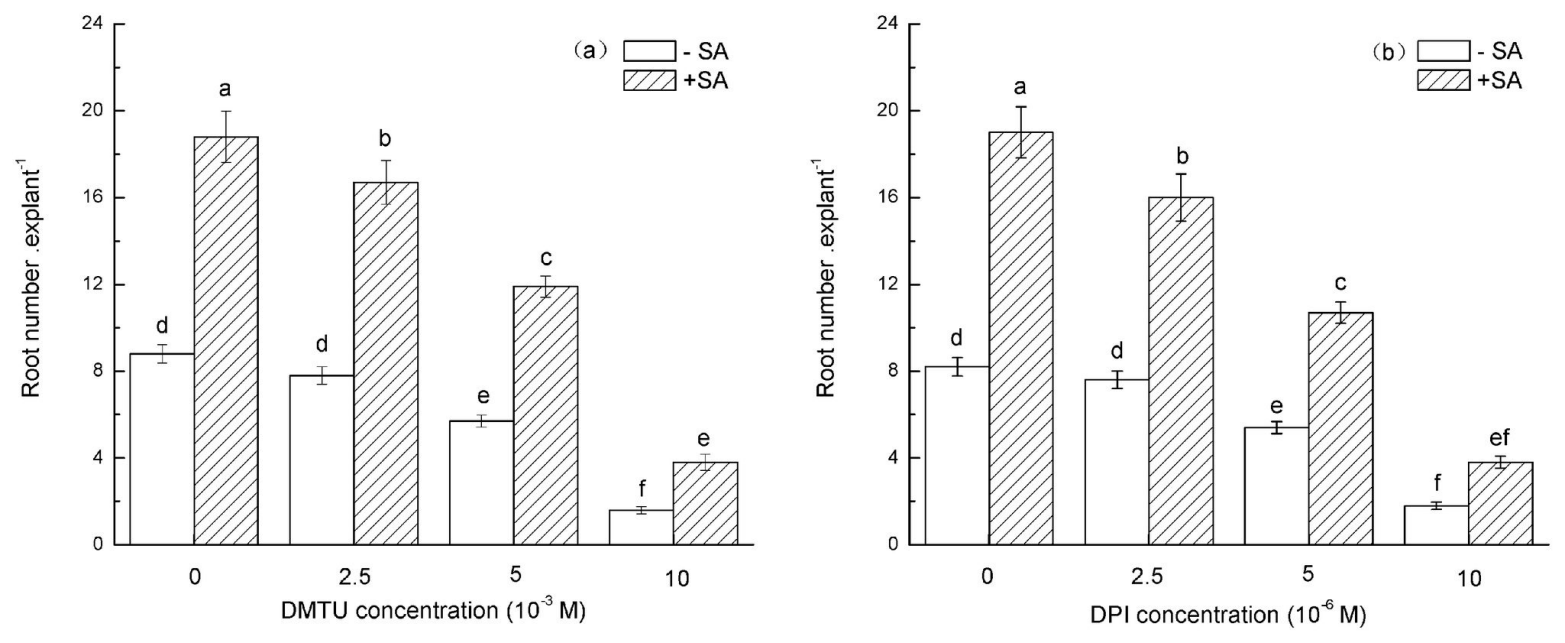
Impact of pre-treatment with DMTU or DPI on SA-induced ARF in mung bean hypocotyl cuttings. The primary roots were removed from seedlings of 5-day-old germinated mung beans, incubated in DMTU or DPI for 4 h, moved into 0.4 mM SA for 24 h, washed three times and cultivated in distilled water for another five days. The number of adventitious roots was quantified and is expressed as the mean from three independent experiments with 30 explants for each treatment. The different letters above the bars indicate significant differences among the treatments (P<0.05), according to the LSD test.

To further elucidate the role of H_2_O_2_ in SA-triggered ARF in mung bean hypocotyls, the role of N, N’-dimethylthiourea (DMTU; a cell-permeable scavenger for H_2_O_2_) [60-64] in mung bean hypocotyls was investigated. We determined that H_2_O_2_ depletion treatment provoked a 2-fold reduction in the root number in comparison with the control explants. We believe that the inhibitory effect of DMTU on ARF can mainly be attributed to the scavenging of H_2_O_2_. However, pretreating hypocotyls with DMTU for 4 h, followed by a transfer into 0.4 mM SA for another 24 h, result indicated that DMTU can partially ameliorate the effect of SA on ARF (Figure 4a). The inhibition of the promotional effect of SA on ARF in mung bean hypocotyls that were pretreated with DMTU indicates that H_2_O_2_ may play a pivotal role in the SA-induced adventitious rooting process. 

The above experiments identified that endogenous H_2_O_2_ might be involved in SA-induced ARF. The production of intracellular H_2_O_2_ was mainly attributable to NADPH oxidase, which converts O_2_ to superoxide anions (O_2_^-^) and then to H_2_O_2_ [65-67]. Diphenyleneiodonium (DPI) is a specific inhibitor of membrane-linked NADPH oxidase that consequently inhibits NADPH oxidase activity (O_2_^-^ synthase) [60,68-71]. The effect of DPI treatment on ARF in mung bean hypocotyls was also investigated. As shown in Figure 4b, pretreatment with DPI alone could depress ARF. We also found that pretreatment with DPI could significantly depress the SA induction of ARF. These results imply that H_2_O_2_ formation might be required for SA-induced ARF. The observed changes in the H_2_O_2_ content after SA treatment further support this conclusion. 

### SA enhances H_2_O_2_ levels in mung bean hypocotyls

**Figure 5:**
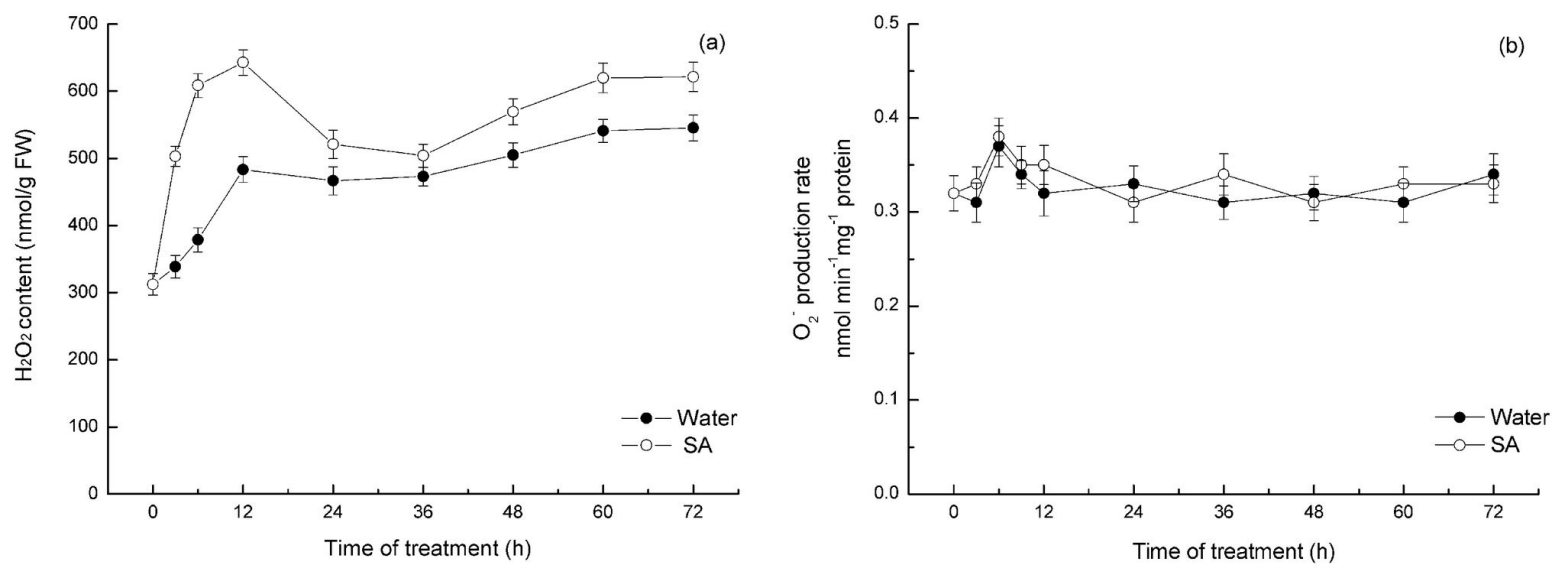
Time course of H_2_O_2_ accumulation (a) and O^2–^ production (b) in the hypocotyls of mung beans treated with water or SA. Explants were incubated with SA or water for 24 h, and the H_2_O_2_ levels were monitored at the indicated time points. The mean values shown are the averages of three different experiments. The error bars represent the SE (n=5). The asterisks indicate that the mean values are significantly different compared with the control values (P<0.05). FW, fresh weight.

To further confirm the interaction between SA and H_2_O_2_ on ARF in mung bean hypocotyl cuttings, we measured the time course of endogenous H_2_O_2_ levels in the hypocotyls of mung beans incubated with water and SA. In this study, SA treatments enhanced H_2_O_2_ levels in the hypocotyls of mung bean seedlings in a time-dependent manner. The treatment of hypocotyls with 0.4 mM SA significantly enhanced in vivo H_2_O_2_ contents compared with hypocotyls treated with water. Here, a rapid increase in the endogenous H_2_O_2_ level was detected during the first 12 h of hypocotyl incubation with SA, with a peak reached at 12 h; however, there was only a slight increase in the H_2_O_2_ level in the water-treated hypocotyls (Figure 5a). The H_2_O_2_ content at 3 h and 6 h was increased by 48% and 61%, respectively, compared with the control seedlings. After 12 h of treatment, the production of H_2_O_2_ reached maximum levels and increased by 33% compared with the control value. The results obtained in this study confirm that SA-mediated ARF has a close association with H_2_O_2_ accumulation. However, the SA treatment did not affect the production of O_2_^–^ throughout the treatment period (Figure 5b).

**Figure 6:**
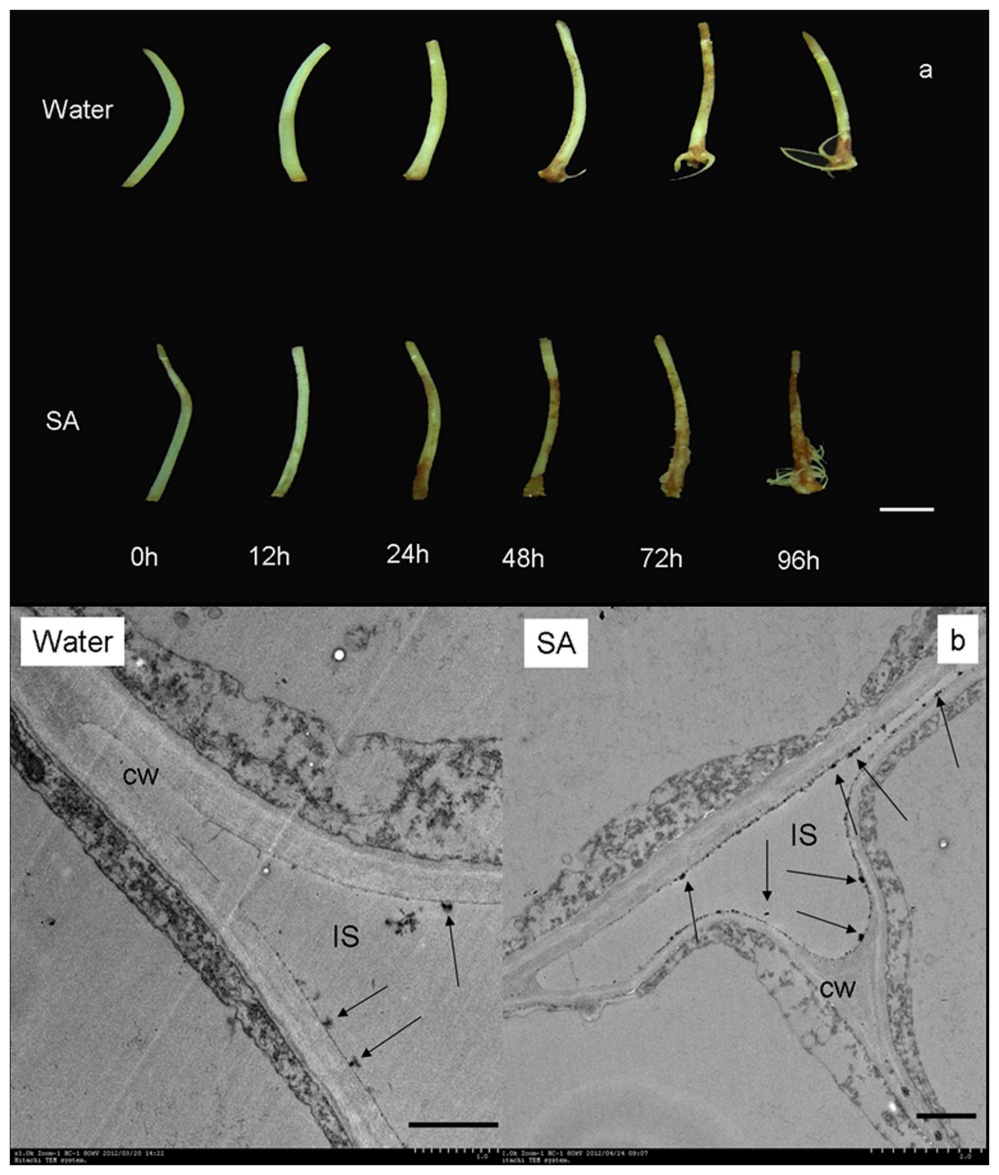
Histochemical and cytochemical detection of H_2_O_2_ accumulation induced by SA in mung bean hypocotyl cuttings. a Mung bean hypocotyl cuttings were incubated in SA for 24 h, and H_2_O_2_ levels were monitored at the indicated time points. All experiments were repeated at least three times with similar results. Bar=1 cm. b Mung bean hypocotyl cuttings were incubated in SA for 12 h. Hypocotyls treated with distilled water under the same conditions served as controls. All experiments were repeated at least three times with similar results. Abbreviations: CW, cell wall; IS, intercellular space. Bar=1 µm.

 In this study, diaminobenzidine (DAB) uptake method has been adapted of *in-situ* detection of H_2_O_2_ [48]. The development of the DAB-H_2_O_2_ reaction product in hypocotyls in response to cutting was shown in Figure 6a. The color was more intense in SA incubated cuttings than water treated seeding, so did the stained area. Histochemical and cytochemical have been used for the detection of H_2_O_2_ generated in mung bean hypocotyls. H_2_O_2_ was visualized at the subcellular level using CeCl_3_ for localization [49]. The greatest accumulation of H_2_O_2_ was observed in the cell wall and intercellular space in SA treated cutting after 12 h incubation, however, there is barely deposits of CeCl_3_ in water-treated seedling (Figure 6b).

### Influence of SA on NADPH oxidase activity and antioxidant enzymes

NADPH oxidase is a protein that transfers electrons from NADPH to an electron acceptor, which leads to the formation of reactive oxygen species. To study a possible link between SA and NADPH oxidase, the activity of NADPH oxidase was measured. Plasma membrane (PM) vesicles were isolated from roots, and the NADPH oxidase activity was determined by measuring O_2_^-^ production. The results indicate that SA treatment did not affect NADPH activity (date not shown) 

**Figure 7:**
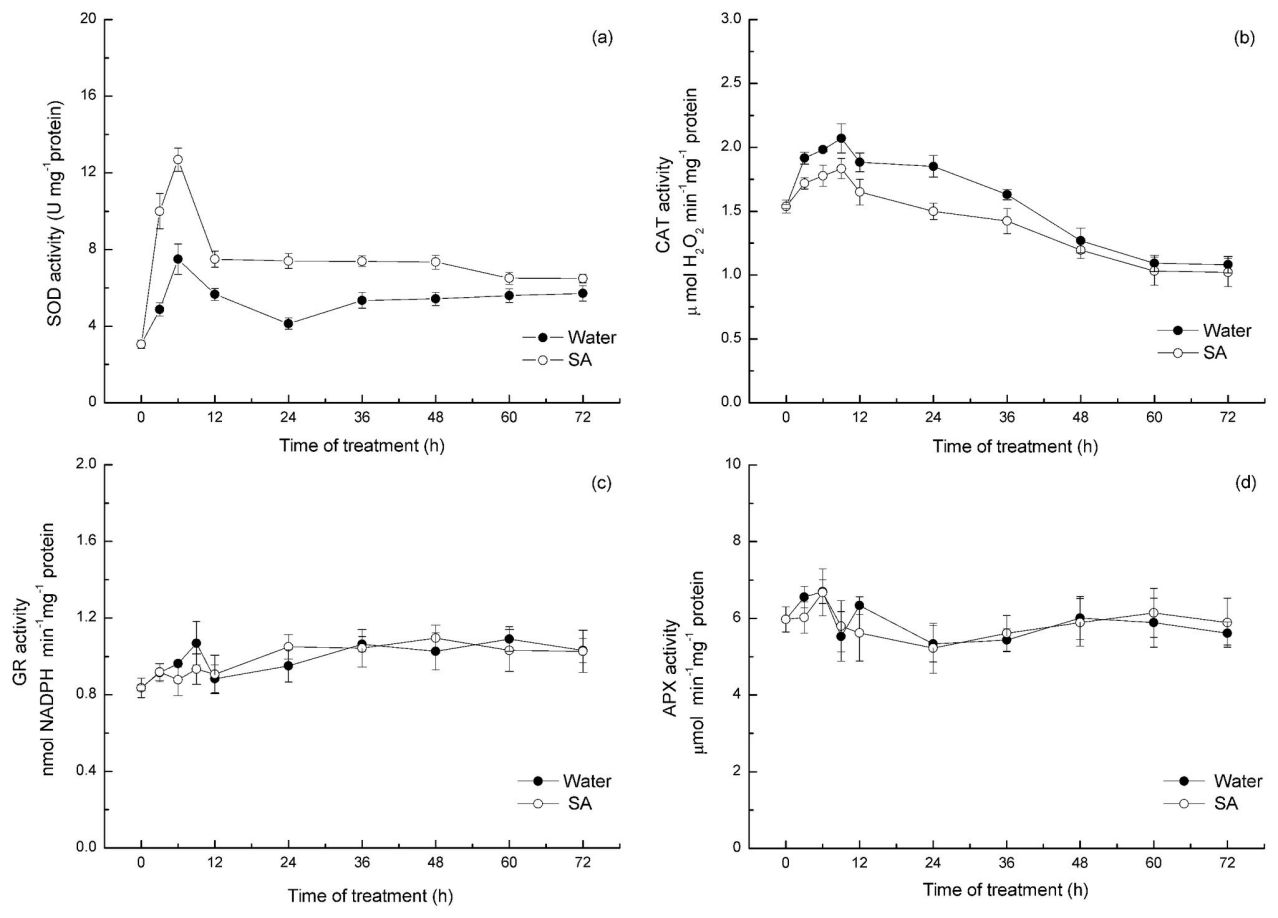
Time course of changes in the activities of the antioxidant enzymes SOD (a), CAT (b), GR(c) and APX (d) in the hypocotyls of mung beans treated with water or SA. Explants were incubated with SA or water for 24 h, and the enzyme actives were monitored at the indicated time point. The mean values shown are the averages of three different experiments. The error bars represent the SE (n=5). Asterisks indicate that the mean values are significantly different compared with the control values (P<0.05). FW, fresh weight.

Altered ROS levels can result either from increased production or decreased scavenging. The activities of the antioxidant enzymes in the cuttings treated with water or SA were investigated to determine the source of H_2_O_2_ formation. As shown in Figure 7a, a significant enhancement in the activity of SOD occurred within the first 6 h of incubation with SA. The SA-induced SOD activity was enhanced by 105% compared with the control value after 3 h of incubation. After 6 h of incubation, the activity of SOD was increased by 69% compared with the control seedlings. It can be presumed that the accumulation of H_2_O_2_ observed with the SA treatment could be a result of SOD activity enhancement. Accordingly, the pretreatment with SA significantly repressed the activities of CAT in a time-dependent manner (Figure 7b). The CAT activities at 3 h and 6 h were decreased by 12% and 15%, respectively, compared with the control seedlings. After 12 h of treatment, the CAT activity was decreased by 17% compared with the control value. However, there was no significant change in APX and GR activities between the water and SA treatments (Figure 7c, 7d).

## Discussion

AR at the base of plant cuttings is an innate de novo organogenesis process that allows the massive vegetative propagation of many economically and ecologically important species [72]. It is necessary to understand the physiological and biochemical process of adventitious rooting. The discovery of signal molecules involved in the intricate network that triggers ARF remains a major goal for a large number of biotechnological procedures. Although a variety of plant components induce ARF and signal transduction has been identified, the molecular mechanism underlying meristem initiation is still undefined and remains to be proven. 

SA is believed to be a key signaling molecular in SAR against pathogens and to play an important role in mediating plant responses to a variety of abiotic stresses. However, the effects of SA in root development are less known. A previous study demonstrated that SA might participate in root formation and development in plants. Some researchers observed that an aqueous solution of SA sprayed on the shoots of soybeans could significantly increase the growth of shoots and roots. In addition, SA induces increases in root growth of up to 100% in the field [73]. Singh [74] observed that SA stimulated root formation in the young shoots of ornamental plants. Salicylic acid and its derivatives are more closely related to these structural requirements. In faba bean (*vicia faba* L.), SA its chemical derivative (acetylsalicylic acid, ASA), at appropriate concentrations could increased rooting efficiency [75]. In Pb^2+^ stress seedlings, SA pretreatment also could significantly increase the length of shoots and roots and partially protect seedlings from Pb^2+^ toxicity [76]. Some researchers have observed that SA promotes later root initiation, emergence and growth, possibly via crosstalk with cytokinin or auxin [77]. However, only a few researchers have noted the role of SA in ARF and the intricate network involving SA and other molecules in this procedure. Our study indicates that SA alone is directly involved in the adventitious rooting process in mung bean hypocotyl cuttings. A dramatic increase of the AR number was observed in SA-treated mung bean hypocotyls cuttings compared with control seedlings (Figure 1a, Figure 2a). The treatments performed with different concentrations of SA confirmed that the effect was dose dependent, with an optimum concentration at 0.4 mM. Those results are consistent with the report of Kling and Riov, who advocated that application of SA increased AR number and yield in mung bean (*Vigna rediata* L.) [5,42]. In addition, SA induced adventitious rooting in a time-dependent manner (Figure 1b). Furthermore, we observed that SA promotes this process through the differentiation of cells to reestablish a new apical meristem (Figure 2b). Accordingly, we confirmed that SA is involved in the adventitious rooting process in mung bean hypocotyl cuttings. 

H_2_O_2_ is a diffusible multifunctional molecule involved in numerous physiological processes in phylogenetically distant species. It is usually regarded as a cytotoxic molecule in cell metabolism. However, there is strong evidence that indicates that H_2_O_2_ is a useful second messenger in plant growth and development and that it mediates various physiological and biochemical processes, including SAR [78,79], stomata closure [80,81], programmed cell death (PCD)[82,83], wounding signaling[49], and root gravitropism [84]. Other researchers have suggested that H_2_O_2_ might possibly be an integral component of intracellular signaling transduction. Guan indicated that H_2_O_2_ plays an important intermediary role in the ABA signal transduction pathway, which leads to the induction of the *Cat1* gene [85]. Pei noted that Ca^2+^-channel activation by H_2_O_2_ is an important mechanism for ABA-induced stomatal closing [71]. H_2_O_2_ also regulates plant cell expansion through the activation of Ca^2+^ channels via a NADPH pathway [86]. In addition, there is also a considerable cross talk between H_2_O_2_ and NO transduction [87].

Increasing evidence supports the notion that H_2_O_2_ plays a causal role in lateral root development and adventitious root formation. Su and others [64] observed that H_2_O_2_ generated by polyamine oxidative degradation is involved in the development of later roots in soybean plants. Furthermore, Li reported that exogenous H_2_O_2_ is an essential component in ARF in mung bean and cucumber hypocotyls cuttings and acts as a second massager in IAA-induced ARF [57,58], yet the mechanisms of H_2_O_2_ in ARF remain elusive.

Early evidence indicates that SA and H_2_O_2_ are involved in the responses of plants to pathogens [88-90]. Although many works have studied the specific interaction between SA and H_2_O_2_ in plants, whether SA and H_2_O_2_ participate in ARF remains unclear. In this study, we observed that SA and H_2_O_2_ played a synergetic role in regulating ARF in mung bean hypocotyl cuttings (Figure 3). The treatment of hypocotyls with SA (SA 0.4 mM) plus H_2_O_2_ (10 mM) resulted in an increased response and an increased root number in comparison with hypocotyls treated with SA or H_2_O_2_ alone. Interestingly, a high level SA and H_2_O_2_ led to a significant suppress in the number of AR. This might be the exacerbation of oxidative stress, resulting in the inhibitory effect on ARF. Accordingly, we can speculate that SA and H_2_O_2_ have additive effect in adventitious rooting process. These results indicate that there is a potential interaction between SA and H_2_O_2_ as signaling molecules. 

However, it is still unclear whether H_2_O_2_ involves signal transduction in relation to the development of SA-induced adventitious rooting. The development of adventitious roots are blocked in hypocotyls treated with DMTU or DPI (Figure 4), implying that endogenous H_2_O_2_ plays a pivotal role in adventitious rooting. These results suggest that DMTU and DPI achieve inhibition via a decrease in the H_2_O_2_ level during the adventitious rooting process. However, when DMTU was used as a trap for H_2_O_2_, the number of SA-induced ARF was significantly reduced, suggesting that SA requires H_2_O_2_ to initiate adventitious rooting. DPI, a specific inhibitor of membrane-linked NADPH oxidase, which is one of the main sources of H_2_O_2_ formation in plant cells, inhibited NADPH oxidase activity and also inhibited ARF in mung bean hypocotyls. Furthermore, the application of a specific concentration of SA alleviated the inhibitory effect of DPI (Figure 4b). The results suggest that H_2_O_2_ may function as a downstream signaling molecule or involved in a parallel pathway in the SA-induced formation and development of AR in mung bean seedlings.

In recent years, considerable evidence has accumulated suggesting that physiologically relevant concentrations of SA treatment can enhance H_2_O_2_ levels, and H_2_O_2_ has been proposed as being functionally downstream of SA in plants based on the evidence that SA can participate in regulating the antioxidant enzymes, such as catalase (CAT), superoxide dismutase (SOD) and ascorbate peroxidase (APX) [61,78,91-94].. In adventitious rooting process, Tewari reported that SA could induce the hydrogen peroxide accumulation in *Panax ginseng* [95]. Furthermore, 40 day-old adventitious roots of *Panax ginseng* treated with 0.2 mM SA could cause an increase in the carbonyl and hydrogen peroxide contents [76]. In contrast, other studies report that SA accumulation could be induced by elevated H_2_O_2_ levels [97,98]. In addition, H_2_O_2_ activates SA biosynthesis via stimulation of BA2H (benzoic acid 2-hydroxylase) activity [97], which is also observed in tobacco cells. Furthermore, other studies have indicated that H_2_O_2_ does not function downstream of SA in regulating PR protein expression [99]. In view of the existing contradictory results derived from in vitro and in vivo studies, the method by which SA enhances H_2_O_2_ production in vivo and the relative significance of such SA-enhanced H_2_O_2_ remains unclear.

However, there is no information, as far as we know, regarding the effects of SA on H_2_O_2_ generated in the adventitious rooting process of mung bean. The interaction between exogenous SA and H_2_O_2_ on ARF in mung bean hypocotyl cuttings was further confirmed by the vivo H_2_O_2_ content determination. The present findings indicate that the enhanced endogenous H_2_O_2_ level was triggered by SA treatment in a time-dependent manner (Figure 5a). A peak H_2_O_2_ content was detected in mung bean hypocotyls incubated with SA for 12 h after the primary root was removed. These results indicate that the rapid increase in H_2_O_2_ content during the first 12 h of incubation could be attributed to the SA stimulus. Nag and others [14] demonstrated that in mung bean hypocotyls cuttings, the induction, initiation and expression phases are 0-24, 24-72 and after 72 h, respectively. We believe that the enhancement of H_2_O_2_ production by SA treatment is useful for cell division and root primordium in the induction phase. Nevertheless, hypocotyl cuttings treated with SA did not present differences in O_2_^-^ content throughout the entire experimental period compared with the water-treated seedlings (Figure 5b). These data suggest that H_2_O_2_ is involved in SA-triggered adventitious rooting, but O_2_^-^ is not. Histochemical and cytochemical method also support the result that SA could induce the H_2_O_2_ accumulation in mung bean hypocotyls. Taken together, these date confirm that H_2_O_2_, and not O_2_^-^, is the limiting factor in SA induced adventitious rooting in mung bean hypocotyls.

These results confirm a tight physiological link between SA and H_2_O_2_ in adventitious rooting process, but the sources H_2_O_2_ of remains to be clarified. In plants, a wide range of abiotic and biotic stresses result in H_2_O_2_ generation from a variety of sources. H_2_O_2_ is removed from cells via a number of antioxidant mechanisms, both enzymatic and nonenzymatic. To further determine the source of H_2_O_2_ enhancement by SA treatment, the activity of NADPH oxidase and the major antioxidant enzymes was also investigated in this paper.

Plasma membrane NADPH oxidase is composed of membrane-bound and cytosolic proteins [100,101]. NADPH oxidase is an enzyme that can use cytoplasmic NADPH to transfer an electron to molecular O_2_ to form O_2_^-^, followed by dismutation of O_2_^-^ to H_2_O_2_ [102,103]. It has been reported that SA treatment was most effective in increasing NADPH oxidase activity in wheat seedlings [104]. To address this issue, we focused our attention on the hypothesis that adventitious root formation induction by SA signals may be involved in the NADPH oxidase pathway. In the end, we observed that there was no difference in the NADPH oxidase activity between the water and SA treatments (date not shown). So, we thought that SA-induced H_2_O_2_ accumulation had no relationship with the NADPH oxidase signal pathway. 

To further investigate the status of the antioxidant defense system in SA-treatment seedlings, the activity of the major ROS-scavenging enzymes, SOD, CAT, APX and glutathione reductase (GR) were investigated. SOD converts highly reactive O_2_^-^ to the more stable molecular-H_2_O_2_. Previous results reported by Rao demonstrated that SA could increase SOD activity in *Arabidopsis thaliana*, which led to increases in the H_2_O_2_ content [61]. In the current paper, higher SOD activity in SA-treated hypocotyls was observed to be time dependent. In addition, the highest SOD activity was observed at 6 h after incubation with SA, and SOD activity increased by 69% compared with the control seedlings (Figure 7a). The enhanced SOD activity in SA-treated hypocotyls might participate in the formation of H_2_O_2_. The existing relationship between SA and SOD activity clearly suggests that hypocotyls treated with SA may have enhanced H_2_O_2_ largely due to the activation of enzymes capable of generating H_2_O_2_. H_2_O_2_ can be removed by CAT or by the APX of the ascorbate-glutathione antioxidant cycle [105]. Previous results indicate that SA inhibits CAT activity and thereby causes an increase in H_2_O_2_ levels [32,78,106]. In the present study, lower CAT activity was detected in SA-treated hypocotyls compared with the water-treated hypocotyls (Figure 7b). These results are in agreement with the hypothesis that SA enhances H_2_O_2_ levels by inactivating the enzymes that are capable of degrading H_2_O_2_. It can be concluded that the accumulation of H_2_O_2_ observed in SA-treated hypocotyls also could be the result of the inhibition of CAT activity. High GR activity maintains the pool of glutathione in the reduced state, allowing GSH to be used by DHAR to reduce DHA to AA [107]. Nevertheless, there was no difference in APX and GR activity between the SA and water-treated cuttings (Figure 7c, 7d). Therefore, APX and GR activity were not the cause of H_2_O_2_ accumulation in the present study. It can be concluded that the accumulation of H_2_O_2_ observed with SA treatment mainly resulted from the enhancement of SOD activity and the inhibition of CAT activity. This mechanism may explain the increase of in vivo H_2_O_2_ content at 12 h after SA incubation. Comparisons in Figure 6 indicate that SA-enhanced H_2_O_2_ levels were related to the increased activities of SOD and were dependent on CAT changes, but that was not the case with GR activity and APX activity. Thus, the conversion of O_2_^-^ by SOD does appear to be the main reason for altered H_2_O_2_ levels.

In conclusion, this study contributes to defining a distinctive role for SA during the adventitious rooting process in mung bean hypocotyls. In addition, hypocotyls treated with SA presented a transient elevation in H_2_O_2_ content. Furthermore, the results of this study indicate that SA enhanced H_2_O_2_ via changes to the status of the antioxidant system. Based on this evidence, we suggest that H_2_O_2_ could be involved in the signaling pathway transduction of SA that triggered ARF in the examined mung bean hypocotyls. However, the underlying mechanism by which SA and H_2_O_2_ induced adventitious root formation requires further investigation.

## References

[1] Geiss G, Gutierrez L, Bellini C (2009) Adventitious root formation: New insights and perspectives. Annual Plant Reviews 37: Root Development

[2] Boyce CK (2005) The evolutionary history of roots and leaves. In: Zwieniecki MA Holbrook NM Vascular transport in plants. Elsevier Amsterdam pp. 479-499.

[3] Jarvis BC, Booth A (1981) Influence of indole-butyric acid, boron, myo-inositol, vitamin D_2_ and seedling age on adventitious root development in cuttings of *Phaseolus* *aureus* . Physiologia Plantarum 81: 213-218.

[4] Eliasson L, Areblad K (1984) Auxin effects on rooting in *pea* *cuttings* . Physiologia Plantarum 61: 293-297. doi:10.1111/j.1399-3054.1984.tb05911.x.

[5] Riov J, Yang SF (1989a) Enhancement of adventitious root formation in mung bean cuttings by 3,5-dihalo -4- hydroxybenzoic acids and 2,4-dinitrophenol. Plant Growth Regulation 8: 277-281. doi:10.1007/BF00025397.

[6] Riov J, Yang SF (1989b) Ethylene and auxin-ethylene interaction in adventitious root formation in *mung* *bean* (Vigna radiata) cuttings. Journal of Plant Growth Regulation 8: 131-141. doi:10.1007/BF02025280.

[7] Sebastiani L, Tognetti R, Paolo P, Vitagliano C (2002) Hydrogen peroxide and indole-3-butyric acid effects on root induction and development in cuttings of *Olea* *europaea* L. Adv Hort Sci 16(1): 7-12.

[8] Theophilus MM, Mibus H, Serek M (2010) The inﬂuence of plant growth regulators and storage on root induction and growth in *Pelargonium* *zonale* cuttings. Plant Growth Regulation 61(2): 185-193. doi:10.1007/s10725-010-9464-y.

[9] Mensuali-Sodi A, Panizza M (1995) Endogenous ethylene requirement for adventitious root induction and growth in tomato *cotyledons* and *lavandin* microcuttings in vitro. Plant Growth Regulation 17: 205-212. doi:10.1007/BF00024727.

[10] McDonald MP, Visser EJW (2003) A Study of the interaction between Auxin and Ethylene in Wild type and transgenic ethylene-Insensitive *Tobacco* during adventitious root formation induced by stagnant root zone conditions. Plant Biology 5: 550-556. doi:10.1055/s-2003-44790.

[11] De Klerk GJ, Hanecakova J (2008) Ethylene and rooting of mung bean cuttings. The role of auxin induced ethylene synthesis and phase-dependent effects. Plant Growth Regulation 56: 203-209. doi:10.1007/s10725-008-9301-8.

[12] Werner T, Motyka V, Strnad M, Schmulling T (2001) Regulation of plant growth by cytokinin. Proceedings of the National Academy of Sciences of the USA 28: 10487-10492. 10.1073/pnas.171304098 PMC56987 11504909

[13] Werner T, Motyka V, Strnad M, Schmulling T (2003) Cytokinin-deficient transgenic *Arabidopsis* plants show multiple developmental alterations indicating opposite functions of cytokinins in the regulation of shoot and root meristem activity. Plant Cell 15: 2532-2550. doi:10.1105/tpc.014928. PubMed: 14555694. 14555694 PMC280559

[14] Nag S, Saha K, Choudhuri MA (2001) Role of auxin and polyamines in adventitious root formation in relation to changes in compounds involved in rooting. Journal of Plant Growth Regulation 20: 182-194. doi:10.1007/s003440010016.

[15] Syros T, Yupsanis T, Zafiriadis H, Economou A (2004) Activity and isoforms of peroxidases, lignin and anatomy, during adventitious rooting in cuttings of *Ebenus* *cretica* L. J Plant Physiol 161: 69-77. doi:10.1078/0176-1617-00938. PubMed: 15002666. 15002666

[16] Li SW, Xue LG, Xu SJ, Feng HY, An LZ (2007) Hydrogen peroxide involvement in formation and development of adventitious root in cucumber. Plant Growth Regulation 52: 173-180. doi:10.1007/s10725-007-9188-9.

[17] Li SW, Xue LG, Xu SJ, Feng HY, An LZ (2009) Hydrogen peroxide acts as a signal molecule in the adventitious root formation of *mung* *bean* seedlings. Environmental and Experimental Botany 65: 63-71. doi:10.1016/j.envexpbot.2008.06.004.

[18] Bellamine J, Penel C, Greppin H, Gaspar T (1998) Confirmation of the role of auxin and calcium in the late phases of adventitious root formation. Plant Growth Regulation 26: 191-194. doi:10.1023/A:1006182801823.

[19] Falasca G, Zaghi D, Possenti M, Altamura MM (2004) Adventitious root formation in *Arabidopsis* *thaliana* thin cell layers. Plant Cell Rep 23: 17-25. PubMed: 15118834. 15118834 10.1007/s00299-004-0801-3

[20] Pagnussat GC, Lanteri ML, Lombardo MC (2002) Nitric oxide is required for root organogenesis. Plant Physiol 129(3): 954-956. doi:10.1104/pp.004036. PubMed: 12114551. 12114551 PMC1540240

[21] Pagnussat GC, Lanteri ML, Lombardo MC (2003) Nitric oxide and cyclic GMP are messengers in the indole aceticacid-induced adventitious rooting process. Plant Physiol 132: 1241-1248. doi:10.1104/pp.103.022228. PubMed: 12857806. 12857806 PMC167064

[22] Pagnussat GC, Lanteri ML, Lombardo MC (2004) Nitric oxidemediates the indole acetic acid induction activation of a mitogen-activated protein kinase cascade involved in adventitious root development. Plant Physiol 135: 279-286. doi:10.1104/pp.103.038554. PubMed: 15122018. 15122018 PMC429373

[23] Lanteri ML, Laxalt AM, Lamattina L (2008) Nitric oxide triggers phosphatidic acid accumulation via phospholipase D during auxin-induced adventitious root formation in *Cucumber* . Plant Physiol 147: 188-198. doi:10.1104/pp.107.111815. PubMed: 18375601. 18375601 PMC2330318

[24] Sorin C, Bussell JD, Camus I (2005) Auxin and light control of adventitious rooting in *Arabidopsis* require argonaute. Plant Cell 17: 1343-1359. doi:10.1105/tpc.105.031625. PubMed: 15829601. 15829601 PMC1091759

[25] Raskin I, Ehmann A, Melader W (1987) Salicylic Acid. a natural inducer of heat production in Arum *lilies* . Science 237: 1545-1549. 10.1126/science.237.4822.1601 17834449

[26] Lee TT, Skoog F (1965) Effect of substituted phenols on bud formation and growth of tobacco tissue culture. Physiologia Plantarum 18: 386-402. doi:10.1111/j.1399-3054.1965.tb06902.x.

[27] Khurana JP, Maheshwari SC (1987) Floral induction in *Wolffia* *microscopica* by non-inductive long days. Plant and Cell Physiology 24(5): 907-912.

[28] Mayers CN, Lee KC, Moore CA (2005) Salicylic acid-induced resistance to *Cucumber* mosaic virus in squash and Arabidopsis thaliana: contrasting mechanisms of induction and antiviral action .Mol Plant Microbe Interact 18(5): 428-434. doi:10.1094/MPMI-18-0428. PubMed: 15915641. 15915641

[29] Anand A, Uppalapati SR, Ryu CM et al. (2008) Salicylic acid and systemic acquired resistance play a role in attenuating crown gall disease caused by Agrobacterium tumefaciens. Plant Physiol 146(2): 703-715. PubMed: 18156296. 18156296 10.1104/pp.107.111302 PMC2245820

[30] Pancheva TV, Popova LP, Uzunova AM (1996) Effect of salicylic acid on growth and photosynthesis in *barley* plants. Journal of Plant Physiology 149: 57-63. doi:10.1016/S0176-1617(96)80173-8.

[31] Khan W, Prithiviraj B, Smith DL (2003) Photosynthetic responses of corn and soybean to foliar application of salicylates. J Plant Physiol 160: 485-492. doi:10.1078/0176-1617-00865. PubMed: 12806776. 12806776

[32] Janda T, Szalai G, Tari I, Paldi E (1999) Hydroponic treatment with salicylic acid decreases the effects of chilling injury in *maize* (*Zea* *mays* *L*.) plants. Planta 208: 175-180. doi:10.1007/s004250050547.

[33] Taşgin E, Atici O, Nalbantoğlu B (2006) Eﬀects of salicylic acid and cold treatments on protein levels and on the activities of antioxidant enzymes in the apoplast of winter wheat leaves. Phytochemistry 67: 710-715. doi:10.1016/j.phytochem.2006.01.022. PubMed: 16519911. 16519911

[34] Munné-Bosch S, Peñuelas J (2003) Photo- and antioxidative protection, and a role for salicylic acid during drought and recovery in field-grown *Phillyrea* *angustifolia* plants. Planta 217(5): 758-766. doi:10.1007/s00425-003-1037-0. PubMed: 12698367. 12698367

[35] Singh B, Usha K (2003) Salicylic acid induced physiological and biochemical changes in wheat seedlings under water stress. Plant Growth Regulation 39: 137-141. doi:10.1023/A:1022556103536.

[36] Borsani O, Valpuesta V, Miguel A (2001) Botella evidence for a role of salicylic acid in the oxidative damage generated by NaCl and osmotic stress in *Arabidopsis* *seedlings* . Plant Physiol 126: 1024-1030. doi:10.1104/pp.126.3.1024. PubMed: 11457953. 11457953 PMC116459

[37] Munns R (2005) Gene sand salt tolerance: bringing them together. New Phytol 167(3): 645-663. doi:10.1111/j.1469-8137.2005.01487.x. PubMed: 16101905. 16101905

[38] Zhang Y, Chen KS, Chen QJ, Zhang SL, Ren YP （2003,) Effects of acetylsalicylic acid (ASA) and ethylene treatments on ripening and softening of postharvest kiwifruit. Acta Botanica Sinica 45 (72): 1447-1452.

[39] Maldonado AM, Doerner P, Dixon RA, Cameron RK (2002) A putative lipid transfer protein involved in systemic resistance signaling in *Arabidopsis* . Nature 419: 399-403. doi:10.1038/nature00962. PubMed: 12353036. 12353036

[40] Ananieva EA, Christov KN, Popova LP (2004) Exogenous treatment with salicylic acid leads to increased antioxidant capacity in leaves of barley plants exposed to paraquat. J Plant Physiol 161(3): 319-328. doi:10.1078/0176-1617-01022. PubMed: 15077630. 15077630

[41] Still SM, Dirr MA, Gartner JB (1976) Phytotoxic effects of several bark extracts on *mung* *bean* and *cucumber* growth. Journal - American Society of Horticultural Science 101(1): 34-37.

[42] Kling GJ, Myer MM (1983) Effects of phenolic compounds and IAA on adventitious root initiation in cuttings of Phaseolus-aureus. Acer HortScience 18(3): 352-354.

[43] Gutierrez L, Mongelard G, Floková K, Pacurar DI, Novák O et al. (2012) Auxin controls *Arabidopsis* adventitious root initiation by regulating jasmonic acid homeostasis. Plant Cell 24(6): 2515-2527. doi:10.1105/tpc.112.099119. PubMed: 22730403. 22730403 PMC3406919

[44] Kang SM, Jung HY, Kang YM, Yun DJ, Bahk JD et al. (2004) Effects of methyl jasmonate and salicylic acid on the production of tropane alkaloids and the expression of PMT and H6H in adventitious root cultures of Scopolia parviﬂora. Plant Sciences 166: 745-751. doi:10.1016/j.plantsci.2003.11.022.

[45] Li L (1995) Effects of resorcinol and salicylic acid on the formation of adventitious roots on hypocotyl cutting of Vigna radiate. Journal of Tropical and Subtropical Botany 3: 67-71.

[46] De Klerk GJ, Guan HY, Huisman P, Marinova S (2011) Effects of phenolic compounds on adventitious root formation and oxidative decarboxylation of applied indoleacetic acid in *Malus* ‘Jork 9’. Plant Growth Regulation 63: 175-185. doi:10.1007/s10725-010-9555-9.

[47] Kordan HA (1985) Endogenous development of adventitious root primordia in *Lettuce* *hypocotyls* . Annals of Botany 55: 267-268.

[48] Thordal-Christensen H, Zhang Z, Wei Y, Collinge DB (1997) Subcellular localization of H_2_O_2_ in plants. H_2_O_2_ accumulation in *papille* and hypersensitive response during the barley-powdery mildew interaction. Plant Journal 11: 1187-1194. doi:10.1046/j.1365-313X.1997.11061187.x.

[49] Orozco-Cárdenas ML, Narváez-Vásquez J, Ryan CA (2001) Hydrogen peroxide acts as a second messenger for the induction of defense genes in tomato plants in response to wounding, systemin, and methyl jasmonate. Plant Cell 13: 179-191. doi:10.2307/3871162. PubMed: 11158538. 11158538 PMC102208

[50] Brennan T, Frenkel C (1977) Involvement of hydrogen peroxide in the regulation of senescence in pear. Plant Physiol 59(3): 411-416. doi:10.1104/pp.59.3.411. PubMed: 16659863. 16659863 PMC542414

[51] Able AJ, Guest DI, Sutherland MW (1998) Use of a new tetrazolium-based assay to study the production of superoxide radicals by tobacco cell cultures challenged with avirulent zoospores of *Phytophthora* *parasitica* *var* *nicotianae* . Plant Physiol 117: 491-499. doi:10.1104/pp.117.2.491. PubMed: 9625702. 9625702 PMC34969

[52] Qiu QS, Guo Y, Dietrich MA, Schumaker KS, Zhu JK (2002) Regulation of SOS1, a plasma membrane Na^+^/H^+^ exchanger in *Arabidopsis* *thaliana*, by SOS2 and SOS3. Proc Natl Acad Sci U S A 99: 8436-8441. doi:10.1073/pnas.122224699. PubMed: 12034882. 12034882 PMC123085

[53] Giannopolitis CN, Ries SK (1977) Superoxide dismutase I. Occurrence in higher plants. Plant Physiol 59: 309-314. doi:10.1104/pp.59.2.309. PubMed: 16659839. 16659839 PMC542387

[54] Aebi H (1984) Catalase in vitro. Methods Enzymol 105: 121-126. doi:10.1016/S0076-6879(84)05016-3. PubMed: 6727660. 6727660

[55] Connell JP, Mullet JE (1986) Pea chloroplast glutathione reductase: puriﬁcation and characterization. Plant Physiol 82: 351-356. doi:10.1104/pp.82.2.351. PubMed: 16665034. 16665034 PMC1056121

[56] Nakano Y, Asada K (1981) Hydrogen peroxide is scavenged by ascorbate-specific peroxidase in spinach chloroplasts. Plant and Cell Physiology 22: 867-880.

[57] Li SW, Xue LG, Xu SJ, Feng HY, An LZ (2007) Hydrogen peroxide involvement in formation and development of adventitious root in cucumber. Plant Growth Regulation 52: 173-180. doi:10.1007/s10725-007-9188-9.

[58] Li SW, Xue LG, Xu SJ, Feng HY, An LZ (2009) Hydrogen peroxide acts as a signal molecule in the adventitious root formation of *mung* *bean* seedlings. Environmental and Experimental Botany 65: 63-71. doi:10.1016/j.envexpbot.2008.06.004.

[59] Li SW, Xue LG (2010) The interaction between H_2_O_2_ and NO, Ca^2+^, cGMP, and MAPKs during adventitious rooting in *mung* *bean* seedlings. In Vitro Cell_Dev_Biol Plant 46(4): 142-148.

[60] Levine A, Tenhaken R, Dixon R, Lamb C (1994) H_2_O_2_ from the oxidative burst orchestrates the plant hypersensitive disease resistance response. Cell 79: 583-593. doi:10.1016/0092-8674(94)90544-4. PubMed: 7954825. 7954825

[61] Rao MV, Paliyath G, Ormrod DP, Murr DP, Watkins CB (1997) Infuence of salicylic acid on H_2_O_2_ production, oxidative stress, and H_2_O_2_-metabolizing enzymes. Salicylic acid mediated oxidative damage requires H 2O _2_. Plant Physiology 115:137-149 10.1104/pp.115.1.137 PMC158469 9306697

[62] Casano LM, Marto AM, Sabater B (2001) Hydrogen peroxide mediates the induction of the chloroplastic *Ndh* complex under photo-oxidative stress in *barley* . Plant Physiol 125: 1450-1458. doi:10.1104/pp.125.3.1450. PubMed: 11244124. 11244124 PMC65623

[63] Jiang MY, Zhang JH (2002) Water stress-induced abscisic acid accumulation triggers the increased generation of reactive oxygen species and up-regulates the activities of antioxidant enzymes in maize leaves. J Exp Bot 53(379): 2401-2410. doi:10.1093/jxb/erf090. PubMed: 12432032. 12432032

[64] Su GX, Zhang WH, Liu YL (2006) Involvement of hydrogen peroxide generated by polyamine oxidative degradation in the development of lateral roots in soybean. Journal of Integrative Plant Biology 48 (4): 426-432. doi:10.1111/j.1744-7909.2006.00236.x.

[65] Alvarez ME, Pennell RI, Meijer PJ, Ishikawa A, Dixon RA et al. (1998) Reactive oxygen intermediates mediate a systemic signal network in the establishment of plant immunity. Cell 92: 773-784. doi:10.1016/S0092-8674(00)81405-1. PubMed: 9529253. 9529253

[66] Potikha TS, Collins CC, Johnson DI, Delmer DP, Levine A (1999) The involvement of hydrogen peroxide in the differentiation of secondary walls in *cotton* ﬁbers. Plant Physiology 119: 849-858. doi:10.1104/pp.119.3.849. PubMed: 10069824. 10069824 PMC32100

[67] Desikan R, Cheung MK, Bright J, Henson D, Hancock JT et al. (2004) ABA, hydrogen peroxide and nitric oxide signaling in stomatal guard cells. J Exp Bot 55: 205-212. PubMed: 14673026. 14673026 10.1093/jxb/erh033

[68] Bolwell GP, Davies DR, Gerrish C, Auh CK, Murphy TM (1998) Comparative biochemistry of the oxidative burst produced by rose and French bean cells reveals two distinct mechanisms. Plant Physiol 116: 1379-1385. doi:10.1104/pp.116.4.1379. PubMed: 9536055. 9536055 PMC35045

[69] Papadakis AK, Roubelakis-Angelakis KA (1999) The generation of active oxygen species differs in *tobacco* and *grapevine* mesophyll protoplasts. Plant Physiol 121: 197-205. doi:10.1104/pp.121.1.197. PubMed: 10482675. 10482675 PMC59368

[70] Orozco-Cardenas ML, Ryan CA (1999) Hydrogen peroxide is generated systematically in plant leaves by wounding and systemin via the octadecanoid pathway. Proceedings of the National Academy of Sciences of the USA 96: 6553-6557. doi:10.1073/pnas.96.11.6553. 10339626 PMC26920

[71] Pei ZM, Murata Y, Benning G, Thomine S, Klüsener B et al. (2000) Calcium channels activated by hydrogen peroxide mediate abscisic acid signalling in guard cells. Nature 406: 731-734. doi:10.1038/35021067. PubMed: 10963598. 10963598

[72] Ramírez-Carvajal GA, Morse AM, Dervinis C, Davis JM (2009) The cytokinin type-B response regulator PtRR13 is a negative regulator of adventitious root development in *Populus* . Plant Physiol 150(2): 759-771. doi:10.1104/pp.109.137505. PubMed: 19395410. 19395410 PMC2689991

[73] Gutierrez-Coronado AG, Lopez CT, Saavedra AL (1998) Effects of salicylic acid on the growth of roots and shoots in *soybean* . Plant Physiol Biochemistry 36 (8): 563- 565. doi:10.1016/S0981-9428(98)80003-X.

[74] Singh SP (1993) Effect of non-auxinic chemicals on root formation in some ornamental plant cuttings. Advances in Horticulture and Forestry 3: 207-210.

[75] Khalafalla MM, Hattori K (2000) Ethylene inhibitors enhance in vitro root formation on faba bean shoots regenerated on medium containing thidiazuron. Plant Growth Regulation 32(1): 59-63. doi:10.1023/A:1006305123585.

[76] Chen J, Zhu C, Sun ZY, Pan XB (2007) Effects of exogenous salicylic acid on growth and H_2_O_2_-metabolizing enzymes in rice seedlings under lead stress. Journal of Environmental Sciences 19: 44-49. doi:10.1016/S1001-0742(07)60007-2. PubMed: 17913152. 17913152

[77] Echevarría-Machado I, Escobedo G, Larque-Saavedra A (2007) Responses of transformed *Catharanthus* *roseus* roots to ferntomolar concentrations of salicylic acid. Plant Physiology and Biochemistry 45: 501-507. doi:10.1016/j.plaphy.2007.04.003. PubMed: 17544287. 17544287

[78] Chen WP, Silva H, Klessig RF (1993) Active oxygen species in the induction of plant systemic acquired resistance by SA. Science 262: 1883-1886. doi:10.1126/science.8266079. PubMed: 8266079. 8266079

[79] Karpinski S, Reynolds H, Karpinska B, Wingsle G, Creissen G et al. (1999) Systemic signaling and acclimation in response to excess excitation energy in Arabidopsis. Science 284: 654-657. doi:10.1126/science.284.5414.654. PubMed: 10213690. 10213690

[80] Lee S, Choi H, Suh S, Doo IS, Oh KY et al. (1999) Oligogalacturonic acid and chitosan reducestomatal aperture by inducing the evolution of reactive oxygen species from guard cells of *tomato* and *Commelina* *communis* . Plant Physiol 121: 147-152. doi:10.1104/pp.121.1.147. PubMed: 10482669. 10482669 PMC59362

[81] Zhang X, Zhang L, Dong F, Gao J, Galbraith DW et al. (2001) Hydrogen peroxide is involved in abscisic acid-induced stomatal closure in *Vicia* *faba* . Plant Physiol 126: 1438-1448. doi:10.1104/pp.126.4.1438. PubMed: 11500543. 11500543 PMC117144

[82] Beers EP, McDowell JM (2001) Regulation and execution of programmed cell death in response to pathogens, stress and developmental cues. Curr Opin Plant Biol 4: 561-567. doi:10.1016/S1369-5266(00)00216-8. PubMed: 11641074. 11641074

[83] Ren D, Yang H, Zhang S (2002) Cell death mediated by MAPK is associated with hydrogen peroxide production in Arabidopsis. J Biol Chem 277: 559-565. PubMed: 11687590. 11687590 10.1074/jbc.M109495200

[84] Joo JH, Bae YS, Lee JS (2001) Role of auxin-induced reactive oxygen species in root gravitropism. Plant Physiol 126: 1055-1060. doi:10.1104/pp.126.3.1055. PubMed: 11457956. 11457956 PMC116462

[85] Guan LM, Zhao J, Scandalios JG (2000) Cis-elements and trans-factors that regulate expression of the maize Cat1 antioxidant gene in response to ABA and osmotic stress: H_2_O_2_ is the likely intermediary signaling molecule for the response. Plant J 22: 87-95. doi:10.1046/j.1365-313x.2000.00723.x. PubMed: 10792824. 10792824

[86] Foreman J, Demidchik V, Bothwell JHF, Mylona P, Miedema H et al. (2003) Reactive oxygen species produced by NADPH oxidase regulate plant cell growth. Nature 422: 442-446. doi:10.1038/nature01485. PubMed: 12660786. 12660786

[87] Neill SJ, Desikan R, Clarke A, Hurst RD, Hancock JT (2002) Hydrogen peroxide and nitric oxide as signalling molecules in plants. J Exp Bot 53: 1237-1247. doi:10.1093/jexbot/53.372.1237. PubMed: 11997372. 11997372

[88] Klessig DF, Malamy J (1994) The salicylic acid signal in plants. Plant Mol Biol 26: 1439-1458. doi:10.1007/BF00016484. PubMed: 7858199. 7858199

[89] Wu G, Shortt BJ, Lawrence EB, Levine EB, Fitzsimmons KC et al. (1995) Disease resistance conferredby expression of a gene encoding H_2_O_2_-generating glucose oxidase in transgenic *potato* plants.Plant Cell 7: 1357-1368. doi:10.2307/3870127. PubMed: 8589621. 8589621 PMC160957

[90] Hammond-Kosack KE, Jones JDG (1996) Resistance gene dependent plant defense responses. Plant Cell 8: 1773-1807. doi:10.2307/3870229. PubMed: 8914325. 8914325 PMC161314

[91] Conrath U, Chen Z, Ricigliano JR, Klessig DF (1995) Two inducers of plant defense responses, 2,6-dichloroisonicotinic acid and salicylic acid, inhibit catalase activities in tobacco. Proceedings of the National Academy of Sciences U_S_A 92: 7143-7147. doi:10.1073/pnas.92.16.7143. PMC41295 11607566

[92] Dat JF, Foyer CH, Scott IM (1998a) Changes in salicylic acid and antioxidants during induced thermo tolerance in mustard seedlings. Plant Physiology 118: 1455-1462. doi:10.1104/pp.118.4.1455. 9847121 PMC34763

[93] Dat JF, Lopez-Delgado H, Foyer CH, Scott IM (1998b) Parallel changes in H_2_O_2_ and catalase during thermo tolerance induced by salicylic acid or heat acclimation in mustard seedlings. Plant Physiology 116: 1351-1357. doi:10.1104/pp.116.4.1351. 9536052 PMC35042

[94] Mateo A, Funck D, Mühlenbock P, Kular B, Mullineaux PM et al. (2006) Controlled levels of salicylic acid are required for optimal photosynthesis and redox homeostasis. J Exp Bot 57 (8): 1795-1807. doi:10.1093/jxb/erj196. PubMed: 16698814. 16698814

[95] Tewari RK, Paek KY (2011) Salicylic acid-induced nitric oxide and ROS generation stimulate ginsenoside accumulation in *Panax* *ginseng* roots. Journal of Plant Growth Regulation 30(4): 396-404. doi:10.1007/s00344-011-9202-3.

[96] Ali MB, Hahn EJ, Paek KY (2007) Methyl jasmonate and salicylic acid induced oxidative stress and accumulation of phenolics in *Panax* *ginseng* bioreactor root suspension cultures. Molecules 12: 607-621. doi:10.3390/12030607. PubMed: 17851415. 17851415 PMC6149333

[97] Leon J, Lawton MA, Raskin I (1995) H_2_O_2_ stimulates salicylic acid biosynthesis in tobacco. Plant Physiology 108: 1673-1678. PubMed: 12228572. 12228572 10.1104/pp.108.4.1673 PMC157549

[98] Chamnongpol S, Willekens H, Moeder W, Langebartels C, Sandermann H et al. (1998) Defense activation and enhanced pathogen tolerance induced by H_2_O_2_ in transgenic *tobacco* . Proc Natl Acad Sci U S A 95: 5818-5823. doi:10.1073/pnas.95.10.5818. PubMed: 9576968. 9576968 PMC20463

[99] Bi YM, Kenton P, Mur L, Darby R, Draper J (1995) H_2_O_2_ does not function downstream of salicylic acid in the induction of PR protein expression. Plant Journal 8: 235-245. doi:10.1046/j.1365-313X.1995.08020235.x. PubMed: 7670505. 7670505

[100] Segal AW, West I, Wientjes F, Nugent JHA, Chavan AJ et al. (1992) Cytochrome b-245 in a ﬂavocytochrome containing FAD and the NADPH-binding site of the microbicidal oxidase of phagocytes. Biochemistry Journal 284: 781-788. 10.1042/bj2840781 PMC1132607 1320378

[101] Desikan R, Burnett EC, Hancock JT, Neill SJ (1998) Harpin and hydrogen peroxide induce the expression of a homologue of gp91-phox in Arabidopsis thaliana suspension cultures. Journal of Experimental Botany 49: 1767-1771. doi:10.1093/jxb/49.327.1767.

[102] Taylor WR, Jones DT, Segal AW (1993) A structural model for the nucleotide binding domains the ﬂavocytochromeb (-245) {beta}-chain. Protein Science 2: 1675-1685. doi:10.1002/pro.5560021013. PubMed: 8251942. 8251942 PMC2142254

[103] Van Gestelen P, Asard H, Caubergs RJ (1997) Solubilization and separation of a plant plasma membraneNADPH-O_2_ ^-^ synthase from other NAD(P)H oxidoreductases. Plant Physiology 115: 543-550. PubMed: 12223822. 12223822 10.1104/pp.115.2.543 PMC158513

[104] Agarwal S, Sairam RK, Srivastava GC, Tyagi A, Meena RC (2005) Role of ABA, salicylic acid, calcium and hydrogen peroxide on antioxidant enzymes induction in *wheat* seedlings. Plant Sciences 169: 559-570. doi:10.1016/j.plantsci.2005.05.004.

[105] Foyer CH, Lopez-Delgado H, Dat JF, Scott IM (1997) Hydrogen peroxide and glutathione- associated mechanisms of acclamatory stress tolerance and signaling. Physiologia Plantarum 100: 241-254. doi:10.1034/j.1399-3054.1997.1000205.x.

[106] Durner J, Klessig DF (1996) Salicylic acid is a modulator of tobacco and mammalian catalases. J Biol Chem 271: 28492-28501. PubMed: 8910477. 8910477 10.1074/jbc.271.45.28492

[107] Noctor G, Arisi ACM, Jouanin L, Kunert KJ, Rennenberg H et al. (1998) Glutathione: biosynthesis, metabolism and relationship to stress tolerance explored in transgenic plants. Journal of Experimental Botany 49: 623-647. doi:10.1093/jxb/49.321.623.

